# Postbiotic gel relieves clinical symptoms of bacterial vaginitis by regulating the vaginal microbiota

**DOI:** 10.3389/fcimb.2023.1114364

**Published:** 2023-02-02

**Authors:** Xin Shen, Lin Xu, Zhiquan Zhang, Yitong Yang, Pengxian Li, Teng Ma, Shuai Guo, Lai-Yu Kwok, Zhihong Sun

**Affiliations:** ^1^ Inner Mongolia Key Laboratory of Dairy Biotechnology and Engineering, Inner Mongolia Agricultural University, Hohhot, Inner Mongolia, China; ^2^ Key Laboratory of Dairy Products Processing, Ministry of Agriculture and Rural Affairs, Inner Mongolia Agricultural University, Hohhot, Inner Mongolia, China; ^3^ Key Laboratory of Dairy Biotechnology and Engineering, Ministry of Education, Inner Mongolia Agricultural University, Hohhot, Inner Mongolia, China; ^4^ Department of Gynecology, Kunming Tongren Hospital, Kunming, Yunnan, China; ^5^ Qingyitang Industrial Co., Ltd., Yunnan, China

**Keywords:** bacterial vaginitis, lactobacilli, *Gardnerella*, vaginal microbiota, postbiotic

## Abstract

Vaginitis is the most common disease in gynecology. Vaginal dysbiosis is a main reason of bacteria vaginitis (BV), as the disrupted microecological environment facilitates the growth of various vaginal pathogens. The most dominant bacteria in the vaginal microbiota are lactic acid bacteria, which are important for maintaining vaginal health. At present, antibiotics and other drugs are often used in clinical treatment, but there are many adverse reactions and easy to relapse, and the intervention of probiotics can help restore vaginal microbiota and alleviate BV. This study is a human clinical trial of 50 patients with bacterial vaginitis (BV). The alleviation effect of applying a postbiotic gel for one week in BV was evaluated. Changes in patients’ clinical indicators of BV (properties of vaginal secretion) and the vaginal microbiota after using the postbiotic gel were monitored. Our results showed that apply the postbiotic gel improved the symptoms of BV, indicated by improvement in the abnormalities of patients’ vaginal secretions. After applying the gel, the relative abundance of vaginal lactobacilli increased compared to baseline. Significant negative correlations were found between lactobacilli and potential vaginal pathogens (including *Gardnerella*, *Prevotella*, and *Atopobium*), as well as the abnormalities of the vaginal secretion. Overall, our results showed that applying the postbiotic gel ameliorated BV, and the symptom improvement was accompanied by significant changes in the bacterial vaginal microbiota. Our study provides valuable clinical data in managing BV.

## 1 Introduction

Vaginitis is one of the most common infectious diseases in female gynecology. It is mainly due to inflammation or infection of the vaginal area, and the clinical symptoms of which include vaginal itching, irritation, and discharge of secretion with unpleasant odor (Walter, 2022). The incidence rate of vaginitis shows an increasing trend due to changes in women’s lifestyle and living habits, such as the use of vaginal lavage and antibiotic application. The vaginal microbiota is known to play an important role in maintaining the health state and homeostasis in the vagina and prevents from vaginitis. Vaginitis is largely related to local infections by pathogens, which is clinically classified into trichomoniasis, mycotic vaginitis, bacterial vaginitis, and vulvovaginal candidiasis (Xiao et al., 2022). Changes in the pH in the vaginal environment are associated with the development of many gynecological diseases. In healthy women, the vaginal environment is weakly acidic, which selects for a specific spectrum of vaginal resident microorganisms, e.g., *Lactobacillus*, that inhibit the growth of some pathogenic bacteria (Onderdonk et al., 2016). However, changes in the pH in the vaginal environment may cause vaginal dysbiosis, and some (opportunistic) pathogens may consume glycogen and inhibit *Lactobacillus*, neutralizing or even alkalizing the vaginal environment and thus favoring the growth of pathogenic bacteria and gynecological disease development.

The structure of the vaginal microbiota comprises mainly lactobacilli, and this group of microbes is thus indicative of vaginal health and homeostatic for the local acidic environment (pH < 4.5) (Oakley et al., 2008; Zhang et al., 2018; France et al., 2022a). Lactic acid is a major metabolite of lactic acid bacteria that is responsible for maintaining the vaginal pH environment. A high content of lactic acid in the vagina enhances the integrity of the epithelial cell barrier, thereby preventing pathogen invasion (Delgado-Diaz et al., 2022). At present, metronidazole and clindamycin are commonly used in treating vaginitis, but antibiotic use may cause various adverse reactions, such as a gradual increase in bacterial drug resistance and a high recurrence rate (Happel et al., 2020). Interestingly, a previous study found that the mechanism of metronidazole for treating BV relies on regulating the vaginal bacterial microbiota (Armstrong et al., 2022); therefore, the vaginal bacterial microbiota could be considered a therapeutic target for improving BV.

Probiotics are active microorganisms that confer beneficial effects on the host (Sun et al., 2022). A growing body of research suggests that probiotics are a safe and effective treatment, especially in gastrointestinal disorders, metabolic disorders, and vaginal inflammatory conditions (Rostok et al., 2019; Schellekens et al., 2021; Wallace et al., 2022). In BV, probiotics can regulate the balance of female vaginal microbiota by promoting the growth of beneficial bacteria while inhibiting the harmful ones. A randomized double-blind controlled trial of an 11-week intervention with a metronidazole vaginal gel with *Lactobacillus crispatus* CTV-05 (Lactin-V) significantly reduced the recurrence rate of BV (Cohen et al., 2020). Another randomized double-blind study of healthy women found that applying a *Lactobacillus*/lactoferrin formulation product could improve the vaginitis symptoms without adverse events (Russo et al., 2018). A recent study transplanted vaginal microbiota from healthy women and applied probiotics in combination could improve the clinical symptoms of BV and restore a healthy vaginal microbiota (Chen et al., 2021). Furthermore, the application of *Limosilactobacillus fermentum* LF15 and *Lactiplantibacillus plantarum* LP01 ameliorates the symptoms of BV, and these exogenously administered probiotics probably integrated into the host vaginal microbiota and adhered to the epithelial cells of the vaginal mucosa, thus establishing physiological protection (Vicariotto et al., 2014). It was found that the inactivated probiotics also had probiotic effects. In 2021, ISAPP issued a consensus statement of postbiotic, which for the first time provided the official concept of postbiotic, that is, postbiotic is a preparation of inanimate microorganisms and/or its components that is beneficial to the health of the host (Salminen et al., 2021). However, at present, there are few reports about postbiotic in vaginitis, and the role of postbiotic in vaginitis is worthy of further exploration.

Clinical identification of BV mainly relies on traditional methods of pap smear and biochemical analysis (Nugent et al., 1991) instead of direct analysis of the vaginal microbiota. However, since most of the vaginal microbiota are anaerobic bacteria, and their nutritional requirements are demanding, traditional culture and isolation identification methods would not be able to provide a full landscape of the vaginal microbiota. Therefore, it would be of interest to apply next-generation technology in identifying and monitoring changes in the vaginal microbiota in relation to symptom improvement. At present, high-throughput sequencing technology has been widely used in analyzing the microbiota in various environments, including food (Wang et al., 2020) and buccal samples (Chen et al., 2019). High-throughput sequencing technology can analyze the composition of vaginal microbiota as a whole, which is an effective approach supplementary to existing biochemical identification methods.

This study recruited 50 patients with BV and investigated the therapeutic effect of one-week intervention with postbiotic gel. Changes in the severity of leucorrhea, biochemical properties and bacterial microbiota of subjects’ vaginal discharge after the gel intervention were analyzed. This study supports that the application of postbiotic gel could improve BV.

## 2 Materials and methods

### 2.1 Trial design and volunteer recruitment

**Figure 1:**
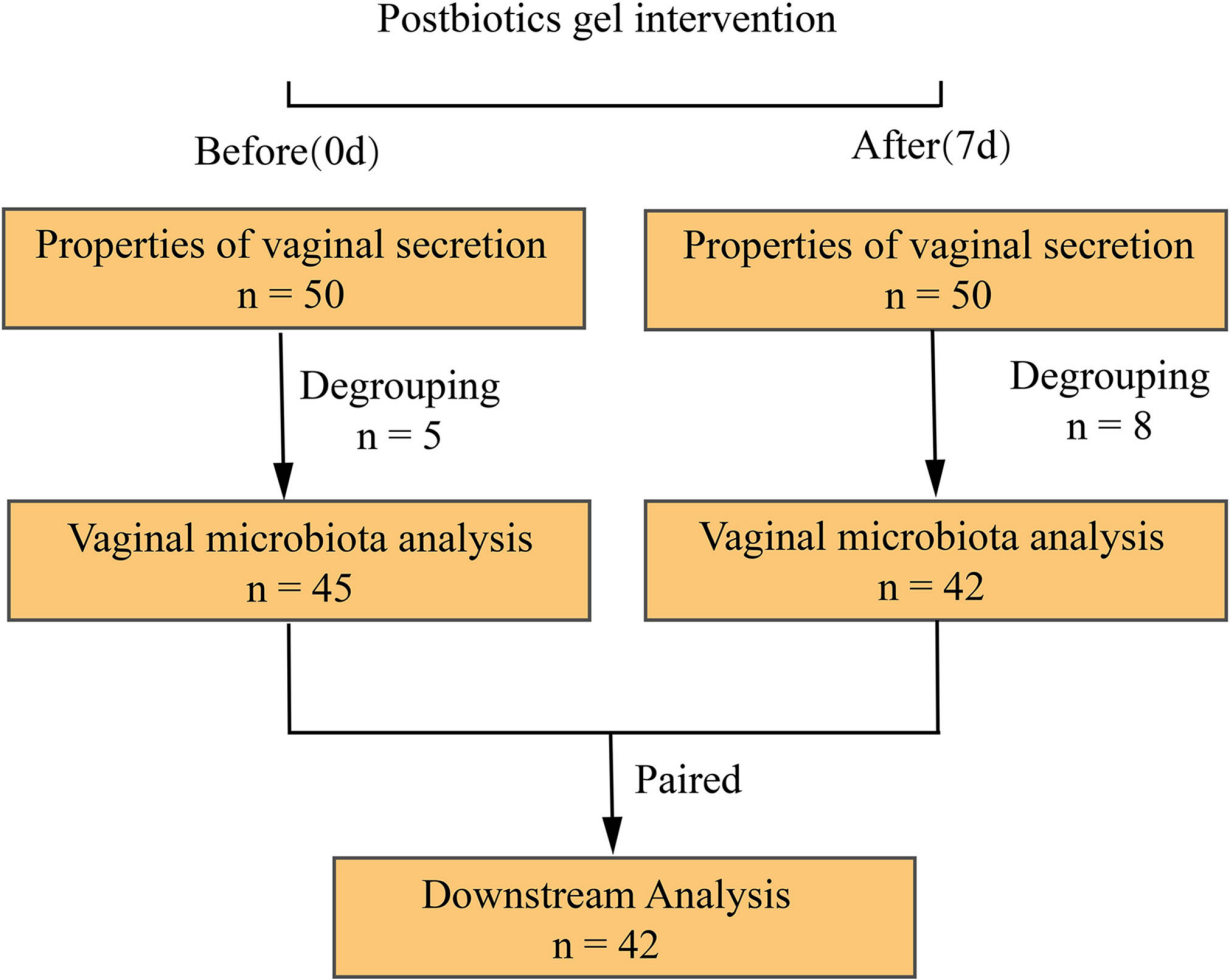
Trial design and enrollment information.

This was a clinical trial conducted between January 2020 to March 2020 at the Department of Obstetrics and Gynecology of Kunming Tongren Hospital. Patients with BV were selected for a one-week intervention of a postbiotic gel to measure changes in the properties of vaginal discharge and vaginal microbiota before (day 0) and after (day 7) intervention. All (n = 50) patients diagnosed with BV at this hospital were considered for participation in the study and were screened by hospital professionals for eligibility. The inclusion criteria were: (1) women clinically diagnosed with diagnostic criteria for BV, aged 18-55 years; (2) body mass index between 19-24; (3) no history of heart, liver, lung, kidney, digestive tract, nervous system, and metabolic abnormalities; (4) not taken other vaginal preparation drugs 30 days before this trial; (5) voluntarily signed the informed consent. Subjects that were allergic to the postbiotics gel were excluded from this study (**Figure 1**).

### 2.2 Postbiotic gel preparation

The postbiotic gel used in this work was produced by Qingyitang Industrial Co., Ltd. (Yunnan, China). Probiotic strains were provided by the Lactic Acid Bacteria Collection Center of Inner Mongolia Agricultural University (Hohhot, China). The gel was prepared by mixing the raw materials (**Table 1**) with distilled water at 60°C, homogenize, sterilize, cool and ferment to pH 4.60 at 37°C. Citric acid was adjusted to pH 4.50 with deionized water. Kabo 940 was added and stirred until completely dissolved, followed by adding and mixing with triethanolamine, propylene glycol, PEG-90M, moisturizing gel, phenoxyethanol, and the fermentation solution in turn. The well-mixed gel was packaged as 3-gram tubes.

**Table 1:** The ratio of raw materials and the amount of probiotics inoculated.

Raw material	Content (%)
Skim milk powder (Fonterra, New Zealand)	5
Full-fat soybean powder (Dragon King of Heilongjiang Agricultural Reclamation)	3
Distilled water	92
*Lacticaseibacillus paracasei* ProSci-92	1×10^6^CFU/mL
*Lacticaseibacillus rhamnosus* ProSci-109	1×10^6^CFU/mL

### 2.3 Postbiotic gel application, collection and analysis of vaginal secretions

Participants was registered to participate in this trial by providing basic demographic information (age: 35.8 ± 8.96) before the start of the trial. The gel was applied every night and avoided the menstrual period. To apply the gel, subjects first cleaned their hands and vulva with warm water, took a product, slightly lifted the hips in a supine position, slowly inserted the gel catheter containing the gel into the deep part of the vagina, pushed it into the pubic area with a pusher, and maintained the posture for about 15 minutes. The product was applied every night for seven days.

Sample collection is carried out by professional doctors. Fresh vaginal secretions were collected with sterile cottons before the start of medication and the next day of the last gel application. In order to obtain fresh samples, we collect the secretions of volunteers according to strict methods and submit them for examination as soon as possible. First of all, sterile cotton swabs were gently rotated in the subjects’ posterior vaginal fornix to collect fresh vaginal secretions. After sampling, cottons with the samples were placed in test tubes containing physiological saline and stored at -80°C until further processing. At the same time, symptoms of subjects, including severity of vulvar itching and leucorrhea, color and odor of vaginal secretions were recorded. The biochemical and microbiological properties of vaginal secretion were determined in the laboratory within 30 minutes of sample collection by a medical professional. Hydrogen peroxide test was performed using a combined aerobic vaginitis/bacterial vaginosis five-item qualitative test kit (based on an enzyme-chemical reaction method; CSC Goldfield Diagnostics, Beijing, China). The presence of lactobacilli, fungi, and the cleanliness of collected samples were observed using a microscope (Olympus Corporation of Japan). The pH of the vaginal secretion was determined using pH test strips. All assessments were operated by professional doctors or personnel, and the data were entered and confirmed by more than two medical staff to ensure data consistency and accuracy. A total of 50 subjects completed the clinical assessment in the trial, and 47 subjects provided a complete sample (two vaginal secretions).

### 2.4 Sequencing of bacterial microbiota in the vaginal samples

The metagenomic DNA in the collected vaginal secretions was extracted by QIAamp kit (Qiagen, Hilden, Germany), and the purity and concentration of the extracted DNA were detected by Nanodrop (Thermo Fisher Scientific, USA) and agarose gel electrophoresis. Qualified DNA samples were amplified, targeting to the 16S rRNA V4 region using barcoded region-specific primers, 515F (GTGYCAGCMGCCGCGGTA) and 806R (GGACTACHVGGGTWTCTAAT). The amplification conditions were pre-denaturation at 95°C for 1 min; denaturation at 95°C for 30 s; annealing at 60°C for 40 s; extension at 72°C for 1 min, for a total of 30 cycles; terminal extension at 72°C for 7 min and termination at 4°C. Amplified samples were checked by agarose gel electrophoresis for the amplicon product size and purity. Follow-up analysis was only performed on amplicon products appearing as a single and bright band.

The samples that met the quality requirement were used for DNA library construction and sequenced using the Illumina novaseq PE250 platform. The original sequences were quality controlled and grouped by the sample nucleotide barcode. Microbial diversity analysis was performed using the QIIME platform (Caporaso et al., 2010) as follows: sequences aligned using PyNAST (Y. Zhang & Alekseyenko, 2017) to establish operational taxonomic units (OTUs) (Vu et al., 2018) according to the UCLUST two-step method. Selected representative sequences were taxonomically assigned by comparing against the SILVA database (Majaneva et al., 2015), the Greengene database (DeSantis et al., 2006), and the Ribosomal Database Project (Release 11.5) database (Maidak et al., 1996).

### 2.5 Statistical analyses

For α diversity analysis, QIIME (version 1.9.1) was used to calculate the Shannon index, chao1 index, and Simpson index. The Shannon curve and the observed species number curve were plotted using R (version 4.2.1), which were used to assess the bacterial diversity of each sequenced sample and the sequencing depth. Wilcoxon tests were used to evaluate the α diversity between samples collected at the two time points. β diversity (weighted and unweighted Unifrac) was calculated by R packages (vegan), and principal coordinate analysis (PCoA) was used for descending presentation. Association between β diversity and study groups was assessed using a non-parametric analysis of similarities (Adonis, vegan R package) with 999 permutations. Followed by comparative analysis to identify significant differential marker bacteria of treatment (cut-off: *P* < 0.05; Wilcoxon test). PICRUST2 (Douglas et al., 2020) was used for functional annotation and STAMP (Parks et al., 2014) was used for data visualization.

## 3 Results

### 3.1 Improvement in subjects’ clinical indicators after postbiotic gel application

In our study, 50 people completed clinical indicators, including 45 people who completed vaginal discharge collection before the intervention and 42 people after the intervention. After matching, the per-protocol population was 42, who completed the process of clinical information collection and provision of vaginal secretion for microbiota sequencing analysis before and after using the postbiotic gel. The vaginal sample of a healthy woman is transparent or milky white, with no odor and in a small amount. A large amount of vaginal secretion that is thin, homogeneous, and of fishy odor is indicative of vaginal inflammation (Amsel et al., 1983). This study compared these properties of subjects’ vaginal secretions before and after use of the postbiotic gel, and found that properties of subjects’ vaginal secretions (e.g., color, clarity, odor) improved significantly after using the postbiotic gel (**Table 2**).

**Table 2:** Changes in vaginal secretion properties and biochemical parameters after the use of postbiotic gel.

Index	Amount of leucorrhea		Leucorrhea traits		Leucorrhea smell		H_2_O_2_		Cleanliness	
	unchanged	19 cases	dilute → thick	10 cases	none → none	45 cases	positive → positive	29 cases	II → II	19 cases
	reduce	29 cases	dilute → thick	1 case	yes → none	5 cases	positive → negative	18 cases	II → III	2 cases
	increase	2 cases	thick → dilute	23 cases			negative → negative	2 cases	III → II	16 cases
			thick → thick	16 cases			negative → positive	1 case	III → III	11 cases
									IV → II	2 cases
Remission rate	64.44%		58.97%		100%		38.30%		62.07%	

Vulvar pruritus is one of the reference indicators for evaluating vaginitis. Three among the 50 patients in this study complaint about vaginal itching at the start of the trial, and the symptom remained only in one subject after using the postbiotic gel. A high level of vaginal secretions cleanliness (the cleanliness rating is closer to IV) and positive reaction in hydrogen peroxide test indicate poor BV or at least poor vaginal health. Initially, 47 patients showed a positive result for hydrogen peroxide, and 18 (remission rate = 38.3%) of them showed a negative hydrogen peroxide result after using the gel. Moreover, 29 people had a cleanliness grade of III or IV, and most (18) of them showed significant improvement after using the postbiotic gel. These data suggested that applying the postbiotic gel for a week could improve BV and vaginal health.

### 3.2 Changes in the α-diversity of vaginal microbiota after the intervention

**Figure 2:**
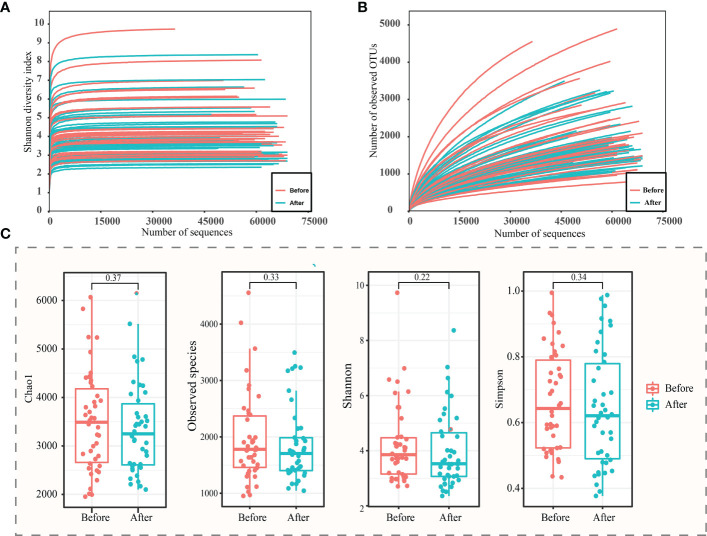
α-diversity analysis of vaginal microbiota before and after the use of the postbiotic gel. Rarefaction curves of **(A)** Shannon diversity and **(B)** observed operational taxonomic units (OTUs). **(C)** Box plots of alpha diversity (chao1, observed species, Shannon and Simpson diversity indexes) before and after using postbiotic gel.

A total of 5,479,608 high-quality sequences were obtained from 87 (Before: n = 45; After: n = 42) sequencing samples. The rarefaction curves of the Shannon diversity (**Figure 2A**), but not the number of observed species (**Figure 2B**) leveled off, suggesting that most of the bacterial diversity was already captured although new species could still be found. Therefore, this sequencing depth was adequate to reflect a representative diversity of the vaginal microbiota.

In order to study the difference between the two groups of samples, the samples were screened, and the samples that could not be paired were excluded, and a total of 42 pairs of samples were obtained. The chao1, number of observed species, Shannon, and Simpson indexes of the vaginal microbiota of subjects were analyzed, and decreasing trends were observed in all four measured diversity indexes after using the postbiotic gel, though the differences were not statistically significant (**Figure 2C**). The decrease in species diversity and abundance could be attributed to the effect of application of the postbiotic gel in shaping the vaginal environment for bacterial growth.

### 3.3 β-diversity analysis, identification of differential bacteria before/after the gel intervention

**Figure 3:**
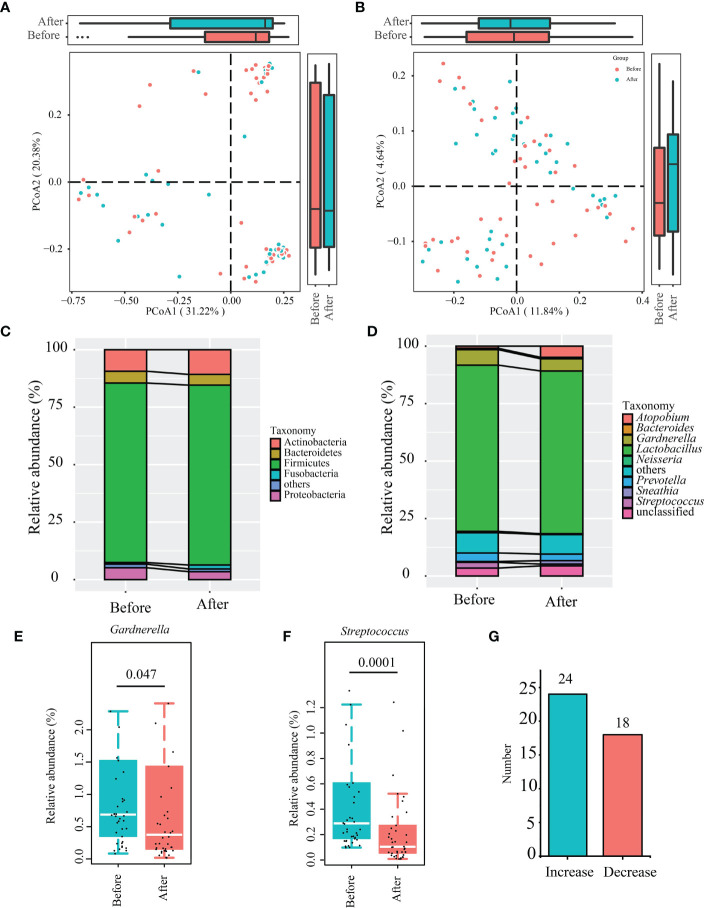
Changes in the β-diversity of vaginal microbiota and differential abundant bacteria identified after using the postbiotic gel. **(A, B)** Principal co-ordinates analysis (PCoA; weighted and unweighted unifrac) of subjects’ vaginal microbiota. **(C)** Phylum- and **(D)** genus-level taxonomic profiles of subjects’ bacterial vaginal microbiota before and after applying the postbiotic gel. **(E, F)** Box plots of the differential abundant bacterial genera, *Gardnerella* and *Streptococcus* before and after using the postbiotic gel. **(G)** The number of people with changes in *Lactobacillus* content.

Changes in the β-diversity of the vaginal microbiota was analyzed by PCoA (weighted and un-weighted Unifrac), which revealed no significant differences before/after the gel intervention (*P* > 0.05; **Figures 3A, B**). The taxonomic analysis of the vaginal microbiota composition revealed five dominant phyla. After applying the gel, the vaginal microbiota showed increased levels in Actinobacteria and Fusobacteria, and decreased levels in Proteobacteria and Bacteroidetes, though the differences were not statistically significant (*P* > 0.05; **Figure 3C**). At the genus level (**Figure 3D**), the major genera (those > 1% average relative abundance before and after using the postbiotic gel) included *Lactobacillus* (71.56%), *Gardnerella* (6.02%), *Prevotella* (3.30%), *Streptococcus* (1.62%), and *Atopobium* (2.89%). After using the postbiotic gels, the relative abundances of some pathogenic bacteria, including *Gardnerella* (**Figure 3E**), *Streptococcus*, and *Prevotella*, decreased, while the relative abundance of *Atopobium* increased. The relative abundance of *Streptococcus* decreased significantly after the gel use (*P* = 0.0001) (**Figure 3F**), while no significant changes were observed in the genus *Atopobium* (*P* = 0.0596; **Table S1**).

We used microscopy to analyze *Lactobacillus* in the secretions and found that *Lactobacillus* was not observed before the gel intervention and 10 samples in which *Lactobacillus* was found after the intervention, but only 5 of these 10 cases were sequenced by Illumina both before and after the use of the gel. Through the analysis of the relative content of *Lactobacillus* in 5 cases, it was found that the relative content of *Lactobacillus* in 4 samples increased in varying degrees. It is worth mentioning that among all sequenced samples (Before: n = 42; After: n = 42), the content of *Lactobacillus* in vaginal secretions of 24 patients increased (**Figure 3G**), accounting for 57% of the total number of patients, it can be seen that exogenous gel can increase the number of lactobacilli vaginal.

### 3.4 Changes in the predicted function of the vaginal microbiota after postbiotic gel application

**Figure 4:**
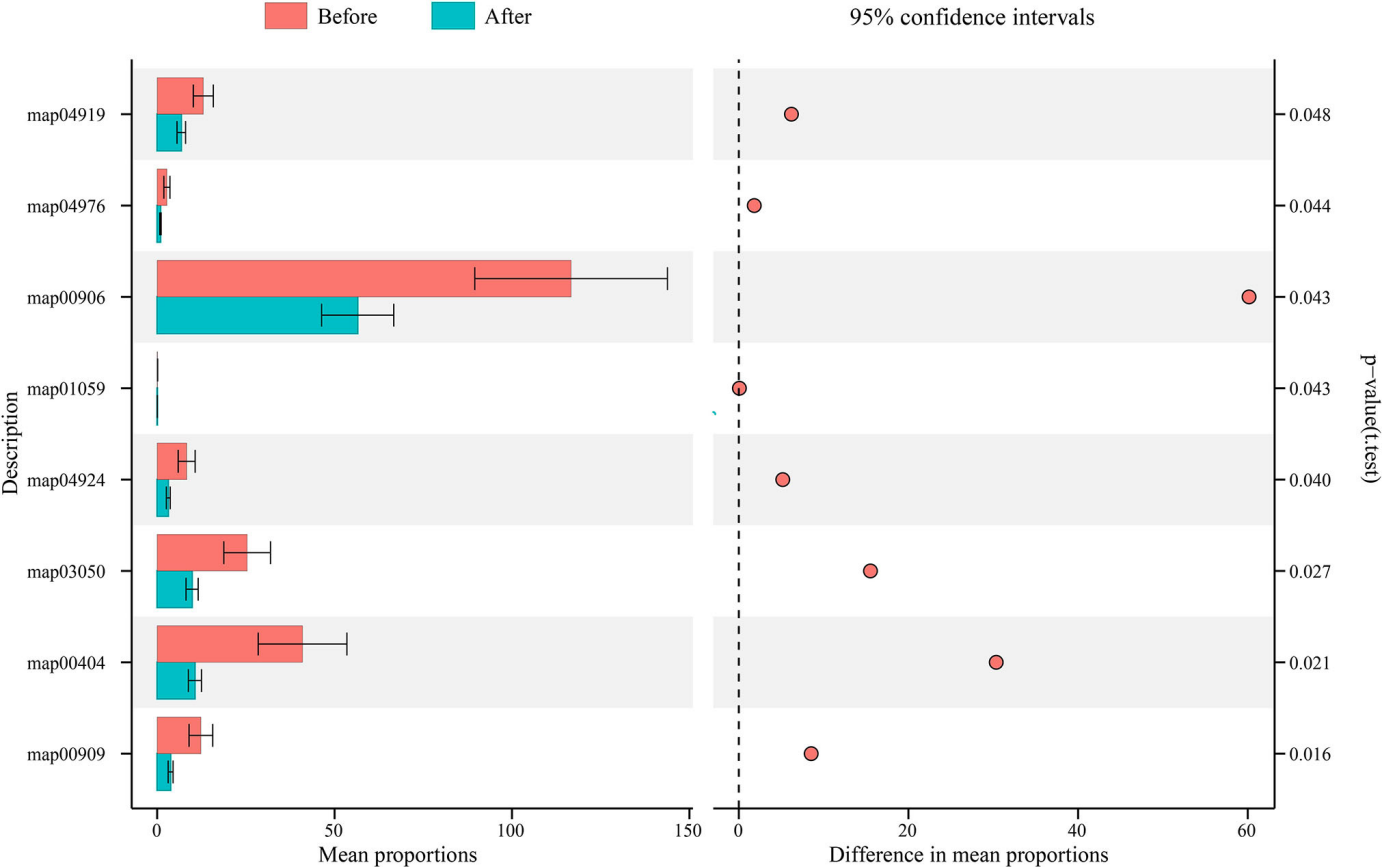
Changes in the functional microbiota predicted by PICRUSt after applying the postbiotic gel. Eight significant differential KEGG pathways (t-test; *P* < 0.05) were identified before (shown in red) and after (shown in blue) applying the postbiotic gel.

We then used PICRUSt2 to predict functional changes in the vaginal microbiota after using the postbiotic gel (**Figure 4**), and significant changes were found in the encoded function of the vaginal microbiota, including pathways of metabolism, genetic information processing, and organic system (**Table S2**). On the secondary level of KEGG pathway, the vaginal microbiota was significantly enriched in the pathways of biosynthesis of other secondary metabolites, metabolism of terpenoids and polyketides, and endocrine system and digestive system, which were significantly decreased after using the gel (*P* < 0.05; **Table S2**). On the tertiary level of KEGG pathway, eight pathways exhibited significant decreases after using the postbiotic gel (*P* < 0.05; **Figure 4**; **Table S2**). These data supported that the application of the postbiotic gel could modulate the predicted functional pathways of the vaginal microbiota, which might be associated with the symptom improvement.

### 3.5 Correlation between dominant microbiota and clinical features of the vaginal secretion

**Figure 5:**
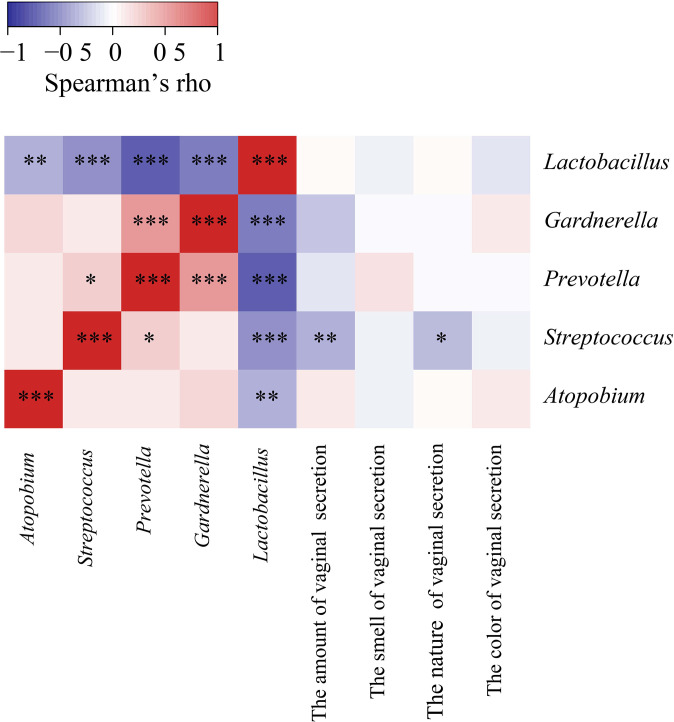
Spearman’s Correlation heat maps showing association between dominant bacteria and properties of subjects’ vaginal secretion. The color scale shows the strength of correlation (ranging from 1 to -1, representing strong positive to strong negative correlation). * *P* < 0.05, ** *P* < 0.01, and *** *P* < 0.001.

According to our clinical observation, we selected the common clinical symptoms and signs, and referred to the symptom scoring standard in the Guidelines for Clinical Research on New Chinese Medicines (for Trial Implementation). For evaluating the amount of secretion: a small amount was scored as 2, a medium amount was scored as 1, and a large amount was scored as 0. For evaluating the nature of secretion: a thin amount was scored as 1, and a thick amount was scored as 0. For evaluating the odor of secretion: an odorless amount was scored as 1, and an odorous amount was scored as 0. For evaluating the color of secretion: a clear color was scored as 3, a light yellow color was scored as 2, a yellow color was scored as 1, and a green color was scored as 0. Then we used Spearman’s correlation analysis to analyze the association between the dominant bacterial genera and clinical features of the collected vaginal samples (**Figure 5**; **Table S3**). Interestingly, we found significant negative correlations between *Lactobacillus* and a number of (potential) pathogens, i.e., *Gardnerella*, *Streptococcus*, *Prevotella*, *Atopobium* (*P* < 0.001, *r* = -0.4937; *P* < 0.001, *r* = -0.4165; *P* < 0.001, *r* = -0.6267; *P* = 0.0043, *r* = -0.3082, respectively). *Streptococcus* showed a weak positive correlation with *Prevotella* (*P* = 0.045, *r* = 0.22), but negative correlations with the amount and characteristics of vaginal secretion (*P* = 0.0061, *r* = -0.2968; *P* = 0.0178, *r* = -0.2579, respectively; **Figure 5**).

## 4 Discussion

The colonization of lactic acid bacteria plays an important role in maintaining vaginal homeostasis by inhibiting other pathogens, and the colonization of anaerobic bacteria is often related to the occurrence of BV (Fredricks et al., 2005). The main reason of the onset of BV is vaginal dysbiosis characterized by a decrease in lactobacilli, leading to the proliferation of pathogenic bacteria such as *Gardnerella vaginalis*, *Prevotella* spp., and *Atopobium vaginae* (Latham-Cork et al., 2021). Antibiotics are traditionally used in treating BV, but antibiotic application can easily disrupt the homeostasis of the vaginal microecology and the endogenous vaginal microflora. Thus, reinfection is common after treating BV with antibiotics. To find a safer and more effective treatment, probiotics have been applied. In this study, we investigated the symptom alleviation effect of applying a postbiotic gel in treating BV. We assessed the changes in the clinical features of vaginal secretion and microbiota in patients with BV after applying postbiotics.

A randomized, double-blind, placebo-controlled trial evaluated the routine treatment of BV or vulvovaginal candidiasis for five days with probiotic capsule, revealing improvement in symptoms, which was accompanied by lactobacilli colonization of the vagina, reduced recurrence rate, and reduced odor in the vaginal discharge, which is consistent with our findings (Ehrström et al., 2010). Furthermore, Cohen et al. (2020) found that applying Lactin-V (a metronidazole vaginal containing *Lactobacillus crispatus* CTV-05) for 11 weeks was effective in reducing the recurrence rate of BV in 228 patients (Cohen et al., 2020). Thus, postbiotic gel is useful in maintaining vaginal health, managing BV, and reduce the recurrent rate.

In this study, we found no significant difference in the α diversity of vaginal microbiota showed a non-significant reduction after using the postbiotic gel. It is important to note that, contrasting to the gut microbiota, a healthy vaginal microbiota has a low bacterial diversity. A high vaginal microbiota diversity increases the probability of BV (Zevin et al., 2016). In addition, the dominance of vaginal lactobacilli is one of the criteria for vaginal health. Consistent to the dominance in lactobacilli observed in this study, Witkin et al. (2013) also showed that the vaginal microbiota comprises a high proportion (71.3%) of *Lactobacillus* (Witkin et al., 2013). Our results showed that applying the postbiotic gel increased the relative abundance of lactobacilli in the vaginal secretion of 24 patients (corresponding to 57% of the total number of patients). Lactobacilli can inhibit the growth of pathogenic microorganisms by producing a variety of secondary metabolites with antibacterial activity, such as lactic acid, hydrogen peroxide, and biosurfactants (Borges et al., 2014). Thus, they play an important role in maintaining the stability of vaginal microenvironment and microbiota.

*Gardnerella* is a conditional pathogen in vaginitis. The overgrowth of *Gardnerella* causes a vaginal dysbiosis, leading to the occurrence of vaginal diseases, such as BV. In the present study, the mean relative abundance of vaginal *Gardnerella* spp. was significantly reduced (from 6.7% to 5.3%; *P* < 0.05) after using the postbiotic gel. *Gardnerella* can adhere to vaginal epithelial cells, forming a dense biofilm, which can enhance the bacterial tolerance to high concentrations of antibacterial molecules like hydrogen peroxide and lactic acid (Hardy et al., 2017). Moreover, the biofilm formation also enhances the resistance of vaginal pathogens to the host mucosal immune defense, favoring problems such as recurrent BV (He et al., 2021). Notably, *Gardnerella* is also the main indicator and pathogen of BV. Therefore, reducing the colonization of *Gardnerella* may be one way to improve BV. The reduction in *Gardnerella* supported that the current probiotic is effective in pathogen inhibition.

Then, a correlation analysis was performed between the dominant genera of the vaginal microbiota and properties of subjects’ vaginal secretions. Again, we found a significant negative association between lactobacilli and potential pathogens like *Gardnerella*, *Atopobium*, and *Prevotella*. It is worth mentioning that BV is often associated with the presence of high loads of *Atopobium vaginae* and/or *Gardnerella vaginalis* (Menard et al., 2008), and that there is a synergistic effect between several bacterial vaginitis pathogens including *Gardnerella*, *Atopobium*, and *Prevotella*. The initial biofilm formed by *Gardnerella* vaginalis in the vaginal epithelium facilitates the attachment and colonization of other pathogenic bacteria, such as *Atopobium*, thus producing more complex biofilms of multiple bacteria, leading to the occurrence of refractory vaginitis (Arroyo-Moreno et al., 2022). In addition, our study found that the predicted function of the vaginal microbiota was improved after using the postbiotics.

In conclusion, our study showed that applying the current postbiotic gel could improve the patients’ symptoms of BV, and the symptom improvement was accompanied by significant changes in patients’ vaginal microbiota, characterized by an increase in lactobacilli and a reduction in multiple potential vaginal pathogens. Our data supported that postbiotics application could improve vaginal health and ease BV. Our findings have a high application value in clinical practice.

## 5 Limitations

There are some limitations in our study. Firstly, the number of subjects included in this trial. was small, and the number of subjects should be increased in subsequent studies with the inclusion of a placebo control group of healthy subjects for a baseline of vaginal microbiota in comparison with subjects with BV. Secondly, 16S rRNA gene amplicon sequencing only represents the genus-level genome without providing any information on gene or protein expression, which would provide more functional information (M. T. France et al., 2022b). In future studies, these aspects should be considered to improve the trial design. Moreover, the trial design should also be expanded to analyze changes in the functional microbiota to provide insights into the mechanism of symptom relief.

## Data availability statement

All sequence data generated in this study were submitted to the MG-RAST database under the ID number mgp95926.

## Ethics statement

The studies involving human participants were reviewed and approved by independent committee members. The patients/participants provided their written informed consent to participate in this study. Written informed consent was obtained from the individual(s) for the publication of any potentially identifiable images or data included in this article.

## Author contributions

XS: Formal analysis, data curation, visualization, writing of the original draft. LX: Clinical trial implementation, specimen collection. ZQZ: Conceptualization, design of methodology. YTY and PXL: Formal analysis, software testing and verification. TM and SG: Supervision of clinical trial, manuscript revision. L-YK: Writing, critical evaluation and revision of the original draft, resource provision. ZHS: Conceived and designed the experiments. All authors contributed to the article and approved the submitted version.

## Supplementary material

Supplementary Table 1 Differential bacterial genera identified before and after using the postbiotic gel.

Supplementary Table 2 Differential pathway identified before and after using the postbiotic gel.

Supplementary Table 3 Spearman correlation analysis between vaginal discharge and differential genera.
