# Supplementary material for: Postbiotic gel relieves clinical symptoms of bacterial vaginitis by regulating the vaginal microbiota
Source: Front Cell Infect Microbiol. 2023 Feb 2;13:1114364. doi: 10.3389/fcimb.2023.1114364 (PMC9936311; doi:10.3389/fcimb.2023.1114364)
Supplement: Supplementary Table 1 — Differential bacterial genera identified before and after using the postbiotic gel. [file Table_1.docx]

**Table S1 Differences between dominant bacterial genera before and after the use of postbiotics gels**

|  | mean, after | mean, before | SD, after | SD, before | Wilcoxon test |
| --- | --- | --- | --- | --- | --- |
| *Streptococcus* | 0.779 | 2.467 | 2.939 | 8.948 | 0.0001 |
| *Gardnerella* | 5.303 | 6.742 | 14.725 | 18.188 | 0.047 |
| *Atopobium* | 4.862 | 0.927 | 14.112 | 3.333 | 0.059 |
| *Neisseria* | 0.353 | 0.604 | 1.539 | 2.874 | 0.279 |
| *Bacteroides* | 0.621 | 0.581 | 2.506 | 1.554 | 0.330 |
| *Sneathia* | 1.469 | 0.387 | 6.179 | 1.534 | 0.542 |
| *Lactobacillus* | 70.805 | 72.306 | 34.819 | 31.611 | 0.687 |
| *Paraprevotella* | 0.001 | 0.001 | 0.003 | 0.003 | 0.971 |
