# Supplementary material for: Postbiotic gel relieves clinical symptoms of bacterial vaginitis by regulating the vaginal microbiota
Source: Front Cell Infect Microbiol. 2023 Feb 2;13:1114364. doi: 10.3389/fcimb.2023.1114364 (PMC9936311; doi:10.3389/fcimb.2023.1114364)
Supplement: Supplementary Table 2 — Differential pathway identified before and after using the postbiotic gel. [file Table_2.docx]

**Table S2 Differential pathway analysis of vaginal microbiota before and after use of postbiotics gel**

| Description | mean, after | mean, before | SD, after | SD, before | *P* value | Description |  |
| --- | --- | --- | --- | --- | --- | --- | --- |
| map00404 | 10.637 | 40.981 | 11.962 | 80.983 | 0.021 | Metabolism;Biosynthesis of other secondary metabolites; Staurosporine biosynthesis | |
| map00906 | 56.510 | 116.664 | 65.877 | 175.917 | 0.043 | Metabolism;Metabolism of terpenoids and polyketides; Carotenoid biosynthesis | |
| map00909 | 3.786 | 12.320 | 4.460 | 21.622 | 0.016 | Metabolism;Metabolism of terpenoids and polyketides; Sesquiterpenoid and triterpenoid biosynthesis | |
| map01059 | 0.044 | 0.113 | 0.048 | 0.210 | 0.043 | Metabolism;Metabolism of terpenoids and polyketides; Biosynthesis of enediyne antibiotics | |
| map03050 | 9.841 | 25.362 | 11.109 | 42.603 | 0.027 | Genetic Information Processing;Folding, sorting and degradation; Proteasome | |
| map04919 | 6.798 | 13.002 | 7.835 | 18.259 | 0.048 | Organismal Systems;Endocrine system; Thyroid hormone signaling pathway | |
| map04924 | 3.131 | 8.313 | 3.564 | 15.457 | 0.040 | Organismal Systems;Endocrine system; Renin secretion | |
| map04976 | 0.920 | 2.740 | 1.282 | 5.532 | 0.044 | Organismal Systems;Digestive system; Bile secretion |  |
