# Supplementary material for: Postbiotic gel relieves clinical symptoms of bacterial vaginitis by regulating the vaginal microbiota
Source: Front Cell Infect Microbiol. 2023 Feb 2;13:1114364. doi: 10.3389/fcimb.2023.1114364 (PMC9936311; doi:10.3389/fcimb.2023.1114364)
Supplement: Supplementary Table 3 — Spearman correlation analysis between vaginal discharge and differential genera. [file Table_3.docx]

**Table S3 Spearman-based calculation of P and R values between dominant bacterial genera and clinical indicators before and after the use of postbiotics gels**

| Index1 | Index2 | P | R |
| --- | --- | --- | --- |
| *Prevotella* | *Lactobacillus* | 1.81E-10 | -0.62671 |
| *Gardnerella* | *Lactobacillus* | 1.82E-06 | -0.49375 |
| *Prevotella* | *Gardnerella* | 1.22E-05 | 0.457467 |
| *Streptococcus* | *Lactobacillus* | 8.13E-05 | -0.41648 |
| *Atopobium* | *Lactobacillus* | 0.004343 | -0.3082 |
| The amount of vaginal secretions | *Streptococcus* | 0.006111 | -0.29682 |
| Characteristics of vaginal secretion | *Streptococcus* | 0.017836 | -0.25795 |
| *Streptococcus* | *Prevotella* | 0.045472 | 0.218872 |
| The amount of vaginal secretions | *Gardnerella* | 0.061017 | -0.20529 |
| *Atopobium* | *Gardnerella* | 0.085483 | 0.188775 |
| The smell of vaginal secretions | *Prevotella* | 0.268171 | 0.122192 |
| The amount of vaginal secretions | *Prevotella* | 0.292971 | -0.11609 |
| The color of vaginal secretions | *Atopobium* | 0.340211 | 0.105351 |
| *Atopobium* | *Prevotella* | 0.381167 | 0.096777 |
| The color of vaginal secretions | *Lactobacillus* | 0.382978 | -0.09641 |
| The amount of vaginal secretions | *Atopobium* | 0.402996 | 0.092435 |
| *Streptococcus* | *Gardnerella* | 0.423249 | 0.088529 |
| *Streptococcus* | *Atopobium* | 0.436006 | 0.086123 |
| The color of vaginal secretions | *Gardnerella* | 0.45524 | 0.08257 |
| The smell of vaginal secretions | *Lactobacillus* | 0.478474 | -0.07839 |
| The smell of vaginal secretions | *Atopobium* | 0.646717 | -0.05074 |
| The color of vaginal secretions | *Streptococcus* | 0.669514 | -0.04725 |
| The smell of vaginal secretions | *Streptococcus* | 0.692362 | -0.0438 |
| Characteristics of vaginal secretion | *Prevotella* | 0.720034 | -0.03969 |
| The amount of vaginal secretions | *Lactobacillus* | 0.735829 | 0.037358 |
| The smell of vaginal secretions | *Gardnerella* | 0.739034 | -0.03689 |
| Characteristics of vaginal secretion | *Atopobium* | 0.740185 | 0.036719 |
| Characteristics of vaginal secretion | *Lactobacillus* | 0.740257 | 0.036709 |
| The color of vaginal secretions | *Prevotella* | 0.742142 | -0.03643 |
| Characteristics of vaginal secretion | *Gardnerella* | 0.97142 | -0.00397 |
